# The intracellular helical bundle of human glucose transporter GLUT4 is important for complex formation with ASPL

**DOI:** 10.1002/2211-5463.13709

**Published:** 2023-09-28

**Authors:** Peng Huang, Hannah Åbacka, Daniel Varela, Raminta Venskutonytė, Lotta Happonen, Jonathan S. Bogan, Pontus Gourdon, Mahmood R. Amiry‐Moghaddam, Ingmar André, Karin Lindkvist‐Petersson

**Affiliations:** ^1^ Department of Experimental Medical Science Lund University Sweden; ^2^ Department of Biochemistry and Structural Biology Lund University Sweden; ^3^ LINXS – Lund Institute of Advanced Neutron and X‐ray Science Sweden; ^4^ Division of Infection Medicine, Department of Clinical Sciences Lund Lund University Sweden; ^5^ Section of Endocrinology and Metabolism, Department of Internal Medicine Yale School of Medicine New Haven CT USA; ^6^ Department of Cell Biology Yale School of Medicine New Haven CT USA; ^7^ Laboratory of Molecular Neuroscience, Division of Anatomy, Department of Molecular Medicine, Institute of Basic Medical Sciences University of Oslo Norway

**Keywords:** adipocyte, ASPL, glucose transporters, GLUT4, trafficking, TUG

## Abstract

Glucose transporters (GLUTs) are responsible for transporting hexose molecules across cellular membranes. In adipocytes, insulin stimulates glucose uptake by redistributing GLUT4 to the plasma membrane. In unstimulated adipose‐like mouse cell lines, GLUT4 is known to be retained intracellularly by binding to TUG protein, while upon insulin stimulation, GLUT4 dissociates from TUG. Here, we report that the TUG homolog in human, ASPL, exerts similar properties, i.e., forms a complex with GLUT4. We describe the structural details of complex formation by combining biochemical assays with cross‐linking mass spectrometry and computational modeling. Combined, the data suggest that the intracellular domain of GLUT4 binds to the helical lariat of ASPL and contributes to the regulation of GLUT4 trafficking by cooperative binding.

## Graphical abstract

Blood glucose levels must be tightly controlled. In fat cells, insulin enhances glucose uptake by stimulating exocytosis of GLUT4 from intracellular compartments resulting in increased GLUT4 levels at the plasma membrane. Here, we show that human GLUT4 binds to ASPL via its intercellular domain to control glucose uptake.

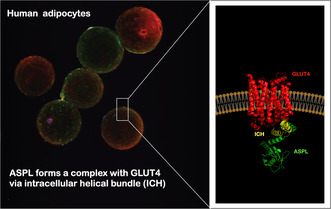

**Abbreviations**
AQP: Aquaporin
ASA: accessible surface area
ASPL: Alveolar Soft Part Sarcoma Locus
ASPL‐C: C‐terminal domain of ASPL
BSA: bovine serum albumin
BSA: buried surface area
CLSM: confocal laser‐scanning microscopy
DM: decyl β‐D‐maltopyranoside
GLUT: Glucose transporter
GSV: GLUT4 storage vesicle
HSA: human serum albumin
ICH: Intracellular helical bundle
ICH_GLUT4_
: GLUT4 intracellular helical bundle
IPTG: isopropyl‐b‐D‐thio‐galactopyranoside
IRAP: insulin‐regulated membrane aminopeptidase
KRH: Krebs‐Ringer HEPES
LC–MS/MS: liquid chromatography tandem mass spectrometry
PFA: paraformaldehyde
PLA: *Proximity Ligation Assay*
RU: response unit
SPR: surface plasmon resonance
TM: transmembrane helix
XL‐MS: cross‐linking mass spectrometry

Glucose is transported from the blood stream across the plasma membranes of cells, utilizing the protein family of glucose transporters (gene symbol *SLC2A*, protein symbol GLUT). The GLUT family is comprised of 14 members (GLUT1‐GLUT14) [1], contributing to the transmembrane flux of several hexoses, such as glucose, fructose, galactose, and mannose, in a concentration dependent manner. GLUTs apply an alternating access mechanism i.e., they undergo cycles of conformational changes between outward‐open and inward‐open states to facilitate transport of hexoses across the membrane [2]. All GLUTs have 12 transmembrane helices spanning the membrane, and two cytosolic termini. In addition, they have an intracellular helical bundle (ICH domain) between transmembrane helix six and seven [3]. GLUT4, encoded by *SLC2A4*, is one of the 14 members of the GLUT family. GLUT4 is an exclusive glucose transporter and the major insulin responsive GLUT in humans [4].

To increase glucose uptake into fat and muscle cells, insulin stimulates the translocation of GLUT4 to the plasma membrane of cells, where it provides influx of glucose from the outside [4]. In unstimulated cells, GLUT4 is sequestered intracellularly in insulin responsive “GLUT4 storage vesicles” (GSVs) [5]. In 2003, it was reported that the protein “tether containing UBX domain for GLUT4” (TUG) is a regulator of GLUT4 trafficking by controlling the intracellular retention and release of GSVs in 3T3‐L1 cells (adipose‐like mouse cell line) [6]. Since then, several publications describing the details of GLUT4‐TUG interaction in rodents have been published (reviewed in [7]), but no structural data or model for this interaction has been reported. Moreover, reports investigating whether a similar setup is present in human tissue and if it contributes to the regulation of GLUT4 trafficking in human adipose tissue in a similar manner, are lacking. The human TUG homolog is the Alveolar Soft Part Sarcoma Locus (ASPL) protein. The sequence identity between human ASPL and mouse TUG is 75% according to clustalw [8] (Fig. S1).

Recently, the cryo‐EM three‐dimensional structure for human GLUT4 was reported, displaying the typical GLUT‐fold [9]. There are no structural data available for the full‐length ASPL; however, the X‐ray structure for the C‐terminal domain of ASPL (residues 317–499) crystallized in complex with the AAA+ ATPase p97, also known as VCP (valosin‐containing protein) is available [10]. Here we report that ASPL is expressed in human white adipose tissue and that it is localized to the cytoplasm. The C‐terminal domain of ASPL was produced at large quantities and confirmed to bind GLUT4 *in vitro*. By performing binding assays, cross‐linking mass spectrometry (XL‐MS) and computational docking, we suggest that GLUT4 forms a complex with ASPL at least in part by utilizing its intracellular helical bundle to bind to the helical lariat of ASPL.

## Results

### ASPL is expressed in human primary adipocytes and forms a complex with GLUT4

**Fig. 1:**
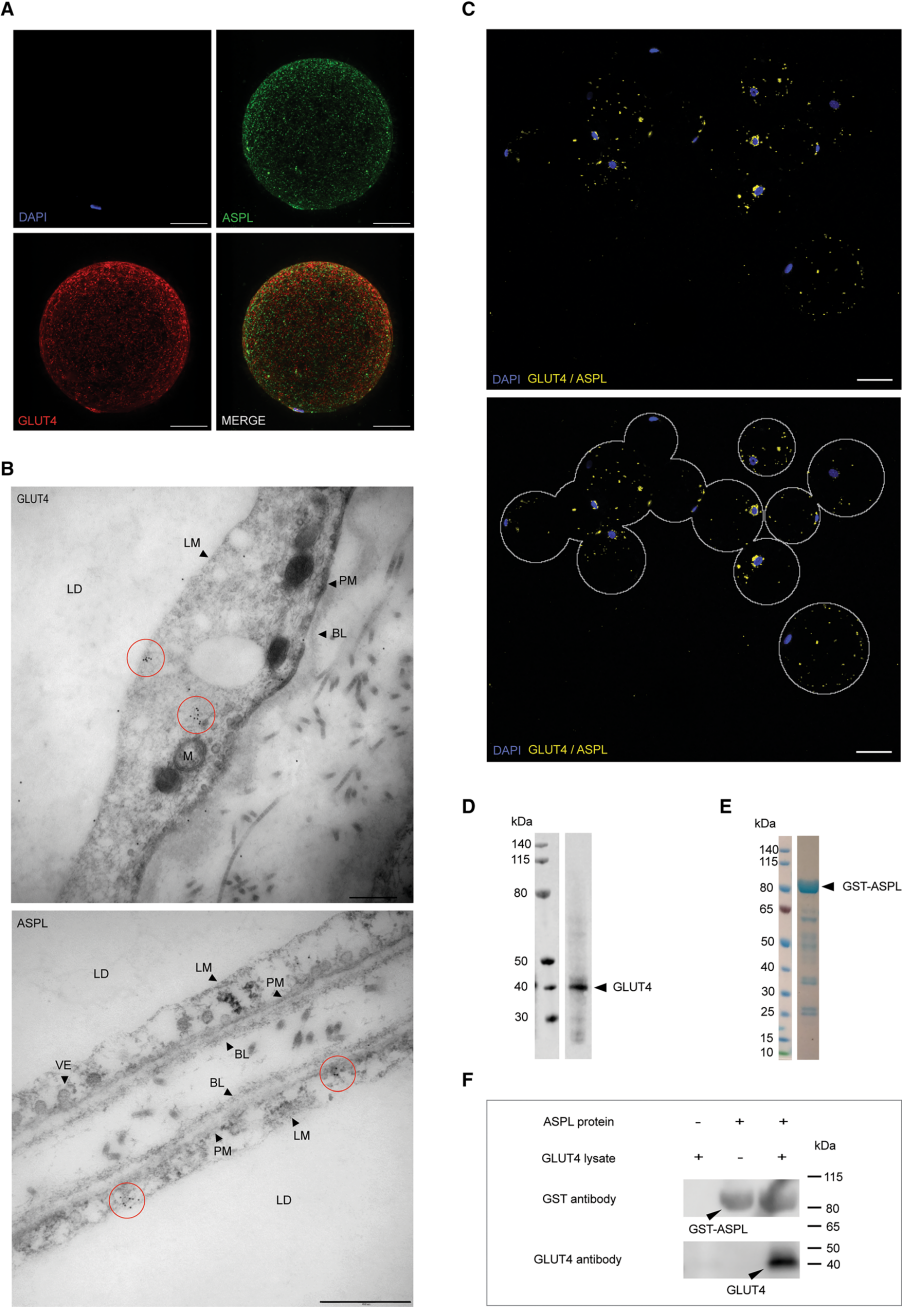
Subcellular localization analysis in human adipocytes and interaction analyses of GLUT4 and ASPL. (A) Confocal microscopy maximum intensity projections of human primary adipocyte with nucleus shown in blue (stained with DAPI), GLUT4 in red and ASPL in green. Magnification is 20× with Nyquist sampling. Scale bars show 30 μm. (B) Gold labeling of GLUT4 (up) and ASPL (down) in human adipose tissue. GLUT4 and ASPL were labeled immunologically by 15 nm gold particles. Gold particle clusters are highlighted with red circles. Scale bars are 500 nm. BL, basal lamina; LD, lipid droplet; LM, lipid monolayer; M, mitochondria; PM, plasma membrane; VE, vesicles. (C) Proximity ligation assay (PLA) of GLUT4 and ASPL in human adipocytes. Close proximity of GLUT4 and ASPL is visualized by yellow dots. Nuclei are stained with DAPI (blue). Microscopy figure shown in original state (up) and with the surface of adipocytes added in gray cartoon (down). Magnification is 20× and scale bars show 50 μm. (D) Western blotting analysis of yeast lysate containing His‐tagged GLUT4 protein. A single band was identified by a specific GLUT4 antibody. (E) Coomassie‐stained SDS/PAGE of purified GST‐ASPL. (F) Pull‐down analysis. Pure GST‐tagged ASPL protein was immobilized on Glutathione 4B beads to capture GLUT4 protein from the yeast lysate.

To investigate if the TUG homolog ASPL is expressed in human adipose tissue and determine its localization, isolated human adipose tissue from female patients with no diagnosed metabolic disorders (type 1 or type 2 diabetes, or thyroid gland dysfunction) were permeabilized and stained using primary ASPL‐specific and GLUT4‐specific antibodies, and the protein distribution was visualized by confocal laser‐scanning microscopy (CLSM; Fig. 1A, Fig. S2). The microscopy analysis showed that both GLUT4 and ASPL were expressed in human primary adipocytes. To obtain a more detailed view of the localization, ultra‐thin sections of human adipose tissue were prepared and stained with gold labeling antibodies of GLUT4 and ASPL. Gold particle clusters representing GLUT4 and ASPL were detected in the cytoplasm of human adipocytes imaged by transmission electron microscopy (TEM), confirming the expression of both GLUT4 and ASPL in the cytoplasm of human adipocytes (Fig. 1B). To further investigate if ASPL and GLUT4 co‐localize, an *in situ Proximity Ligation Assay* (PLA) was executed. PLA is a technique that enables determination of close proximity interactions between proteins [11]. Unlike conventional immunofluorescence co‐localization analysis based on co‐distribution of fluorescent probes, *in situ* PLA depends on the close proximity of two adjacent proteins for signal generation, and thus this method is suitable for protein interaction studies. Briefly, the two proteins of interest are labeled using protein specific primary antibodies (here ASPL and GLUT4) and subsequently with a set of proximity probes *i.e*. secondary antibodies with a single‐stranded oligonucleotide sequence. Only if the two proximity probes are brought close together (*i.e*. a protein complex), a double stranded DNA circle will be generated by ligation and hybridization, and be seen as distinct fluorescent spots [11]. As can be seen in Fig. 1C, distinct spots are detected which are especially prominent around several of the nuclei (Fig. 1C). To investigate if GLUT4 interacts with ASPL equivalently to GLUT4 and TUG in rodents, a pull‐down assay was performed. Briefly, yeast lysate expressing GLUT4 (Fig. 1D) was applied to beads immobilized with pure and homogeneous ASPL protein fused with GST (Fig. 1E). When no ASPL is bound to the beads, GLUT4 is not captured and thus not eluted, while if ASPL is bound to the beads, GLUT4 is captured and subsequently eluted (Fig. 1F). This demonstrates that GLUT4 can form a complex with ASPL.

### ASPL interacts with GLUT4 utilizing its C‐terminal domain

**Fig. 2:**
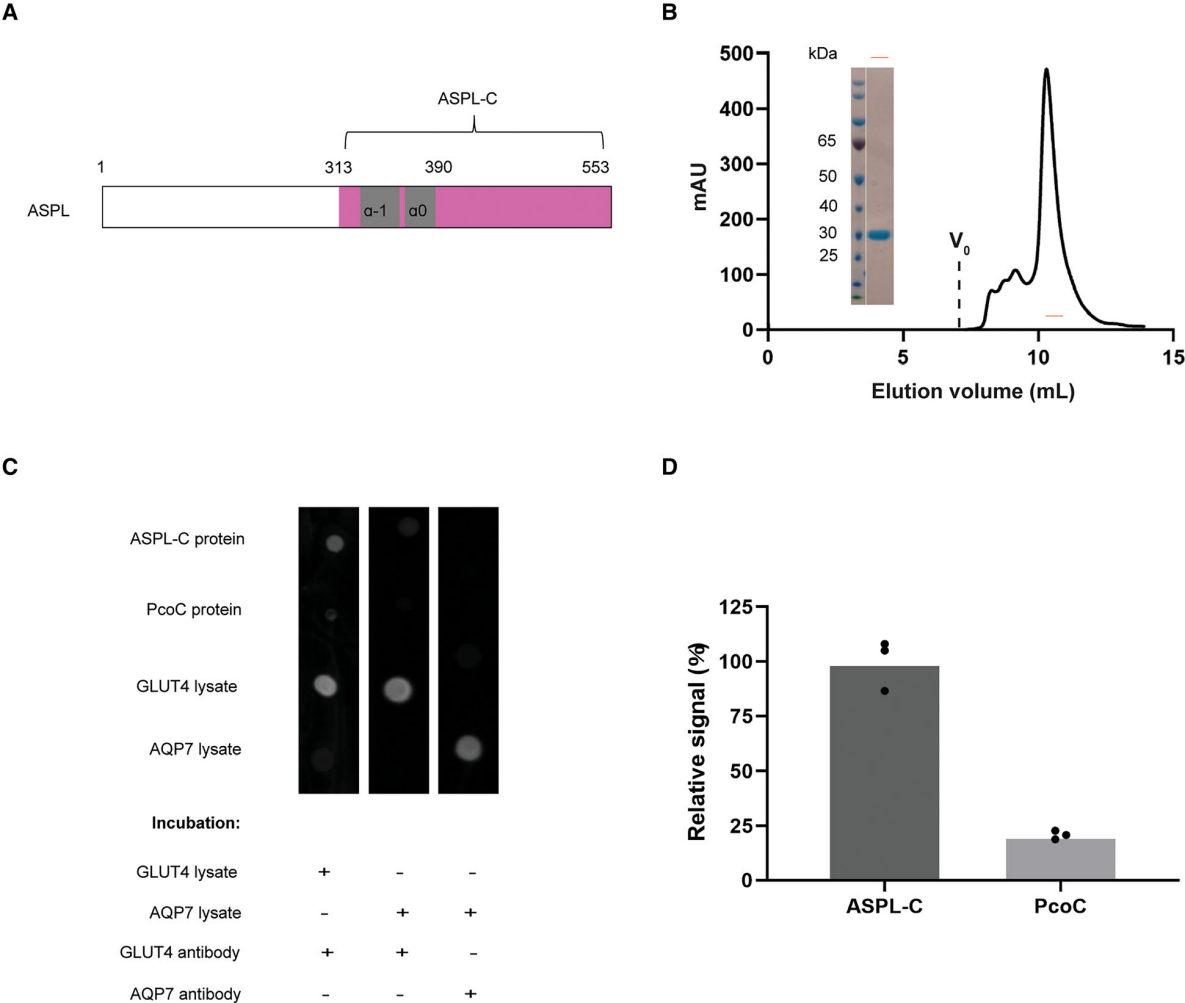
GLUT4 interacts with the C‐terminal domain of ASPL. (A) Schematic diagram of ASPL with C terminus (ASPL‐C) colored by magenta and the two helices in the helical lariate colored by gray. (B) Size‐exclusion chromatography (SEC) profile and coomassie‐stained SDS/PAGE of purified ASPL‐C. (C) Representative far dot western blot analysis with immobilized ASPL‐C protein, PcoC protein (as a control) GLUT4 or AQP7 lysate, incubated with GLUT4 and AQP7 lysate (as control lysate), respectively prior to detection with GLUT4 or AQP7 antibody. (D) Quantification analysis of immunosignals on dot blots. The relative binding signal of ASPL‐C and PcoC upon GLUT4 is shown as the mean of three replicates.

Previously the C‐terminal domain of ASPL (ASPL‐C; Fig. 2A) in complex with p97 has been structurally determined by X‐ray crystallography [10]. To investigate if the C‐terminus of ASPL contributes to the complex formation with GLUT4, ASPL‐C was overexpressed in *Escherichia coli* and purified to homogeneity (Fig. 2B). A far dot western blot approach was used to study the interaction between GLUT4 and ASPL‐C. This method is superior to the conventional far western blot when the interaction is dependent on the native three‐dimensional structure of the proteins. Briefly, the ASPL‐C, PcoC (a protein related to bacterial copper homeostasis, used as a negative control) or, for additional validation, yeast lysates containing either GLUT4 or an aquaporin, AQP7, were spotted onto membranes and exposed to the GLUT4 or AQP7 yeast lysates. The potential binding of GLUT4 to ASPL‐C was detected with a GLUT4 specific antibody. Firstly, when incubating the membrane with GLUT4 lysate, a strong signal was detected where ASPL‐C was spotted with the GLUT4 specific antibody (Fig. 2C). Likewise, a clear signal was also detected where the GLUT4 lysate was spotted, confirming that the lysate contains GLUT4. Secondly, repeating the experiments with negative control lysate (expressing AQP7) prior to immunostaining with GLUT4 antibody, no signal was detected where either ASPL‐C or control lysate was spotted, but still a clear signal was detected where the GLUT4‐lysate was spotted, confirming the specificity of the antibody (Fig. 2C). Quantification of the immunosignals on the dot blots further corroborates the interaction between GLUT4 and the C‐terminal domain of ASPL (Fig. 2D).

### ASPL‐C interacts with GLUT4 intracellular helical bundle (ICH)

**Fig. 3:**
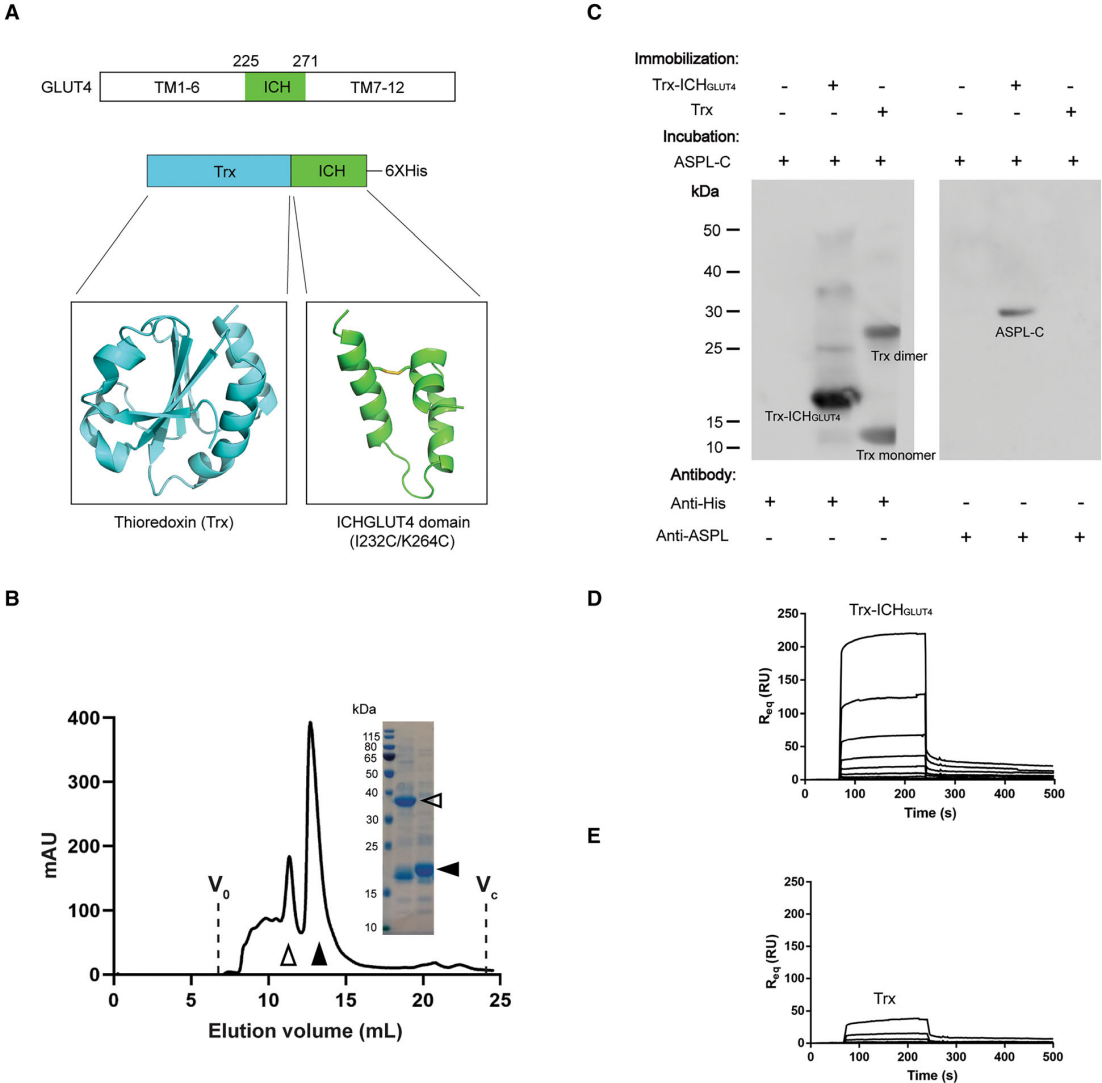
GLUT4 interacts with ASPL‐C by intracellular helical domain. (A) Schematic diagram of GLUT4 with ICH domain colored by green. Cartoon representation of Trx‐ICH_GLUT4_ construct. (B) Size‐exclusion chromatography (SEC) profile and Coomassie‐stained SDS/PAGE of purified Trx‐ICH_GLUT4_. Dimer and monomer of Trx‐ICH_GLUT4_ are illustrated by empty and solid arrows, respectively. (C) Pull down assay for Trx‐ICH_GLUT4_, Trx and ASPL‐C. Trx‐ICH_GLUT4_ or Trx was immobilized on nickel resin before incubation with ASPL‐C protein, and co‐eluted proteins were detected by anti‐His antibody and anti‐ASPL antibody, respectively. ASPL‐C was immobilized on the surface of a CM5 chip, and a two‐fold concentration series of (D) Trx‐ICH_GLUT4_ and (E) Trx ranging from 0.6 μm to 83 μm were injected, respectively. The responses (response unit, RU) were recorded and shown against time (s).

It has previously been suggested that the intracellular helical bundle (ICH) of GLUT4 located between transmembrane helix 6 (TM6) and transmembrane helix 7 (TM7; Fig. 3A) is important for the interaction with TUG [12]. To clarify if this domain also contributes to the complex formation with ASPL, the GLUT4 intracellular helical bundle (ICH_GLUT4_) including residues 225–271 and I232C/K264C substitution to facilitate structural stability of the isolated helical bundle via disulphide bond formation, was fused to the C‐terminus of thioredoxin (Trx; Fig. 3A), expressed in *E. coli* and purified to homogeneity (Fig. 3B). The Trx‐ICH_GLUT4_ was eluted as two peaks from the size‐exclusion column corresponding to molecular weight of approximately 36 and 18 kDa, respectively, thus likely forming dimers and monomers. The monomer protein was selected for further studies (Fig. 3B). Trx or Trx‐ICH_GLUT4_ was bound to Ni‐NTA beads via an introduced His‐tag and ASPL‐C was applied. Upon elution, ASPL signal was only detected when co‐eluted with Trx‐ICH_GLUT4_, but not with His‐tagged Trx, showing that ICH_GLUT4_ contributes to the interaction with ASPL‐C (Fig. 3C). To further confirm this interaction, surface plasmon resonance (SPR) was carried out. ASPL‐C was immobilized on the CM5 chip and Trx or Trx‐ICH_GLUT4_ were applied as analytes. Concentration series of Trx or Trx‐ICH_GLUT4_, ranging from 0.6 to 83 μm, were injected over the immobilized ASPL‐C and the responses were recorded (Fig. 3D). A concentration dependent increase in response for Trx‐ICH_GLUT4_ was detected, confirming that the GLUT4 intracellular helical bundle most likely contributes to the complex formation between ASPL‐C and GLUT4 (Fig. 3D). A significantly lower response was observed for Trx, although some increase in response was detected for the two highest concentrations tested (Fig. 3E). To further evaluate the affinity between Trx‐ICH_GLUT4_ and ASPL‐C, the K_D_ was calculated, excluding the two highest concentrations based on the observed background binding to Trx, to approximately 70 μm although this value needs to be further validated as no saturation was observed for the binding curves (Fig. S3).

### Cross‐linking mass spectrometry‐based analysis of the complex between ASPL‐C and ICH_GLUT4_

**Fig. 4:**
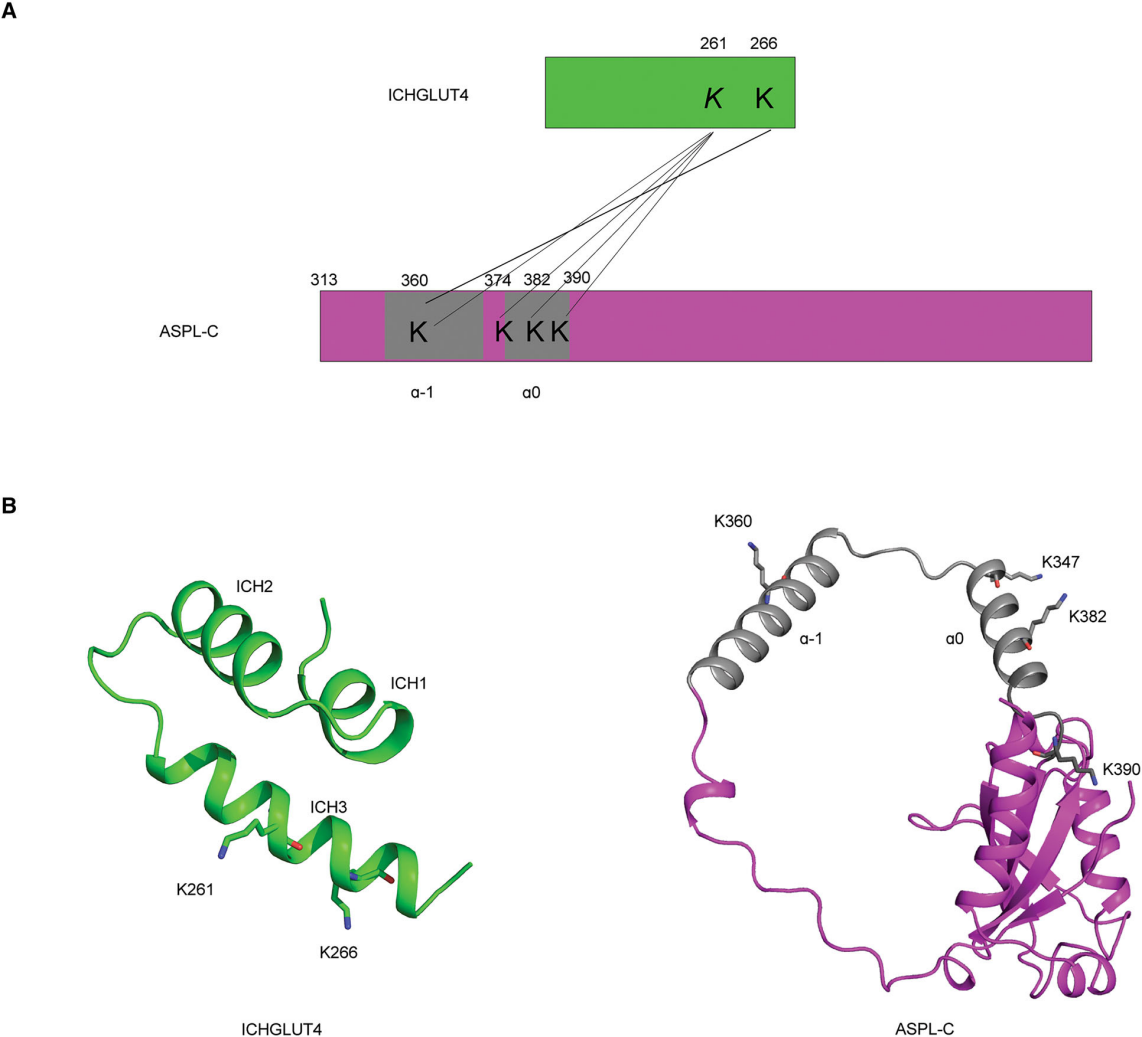
Cross‐linking mass spectrometry (XL‐MS) analysis for ICH_GLUT4_ and ASPL‐C. (A) Schematic diagram of crosslinking sites between ICH_GLUT4_ and ASPL‐C connected by black lines in pairwise. (B) The cartoon representation of ICH_GLUT4_ (green), ASPL‐C (magenta with the helical lariat in gray), and lysines contributing to the complex formation in sticks.

To obtain distance constraints for structural modeling of the ICH_GLUT4_ and ASPL‐C complex, Trx‐ICH_GLUT4_ (18 kDa) was cross‐linked with ASPL‐C (26 kDa) using disuccinimidyl suberate (DSS). All samples were proteolytically digested for liquid chromatography tandem mass spectrometry (LC–MS/MS) analysis. Analysis of the data identified five groups of intermolecular cross‐linking sites between ICH_GLUT4_ and ASPL‐C, excluding those located in the Trx (Fig. 4A and Table 1). Interestingly, all cross‐linked lysines at ICH_GLUT4_ are on the ICH3 helix, while the ones at ASPL are localized to the so‐called helical lariat with a lasso shape, mainly at the α_0_ helix (Fig. 4B), which correlates well with previously published data where residues 313–376 of TUG are shown to be necessary for TUG‐C to bind GLUT4 [6].

**Table 1:** Crosslinking sites between ICH_GLUT4_ and ASPL‐C identified by mass spectrometry and crosslinking residues distance in model 3 and 5.

ICH_GLUT4_	ASPL‐C	Best linkage position on ICH_GLUT4_	Best linkage position on ASPL‐C	Residues distance in model 3 (Å)	Residues distance in model 5 (Å)
KLERER	LAQLKSER	K266	K360	26.5	20.8
LTGWADVSGVLAELKDECR	EAQIKEK	K261	K382	14.6	15.6
LTGWADVSGVLAELKDECR	LAQLKSER	K261	K360	27.6	27.7
LTGWADVSGVLAELKDECR	LEEAPLVTKAFR	K261	K374	9.5	20.7
LTGWADVSGVLAELKDECR	YPKVALR	K261	K390	28.2	24.5

## Discussion

Few physiological parameters are more tightly and acutely regulated in humans than blood glucose levels. TUG plays an important role in the regulation of glucose homeostasis by controlling the GLUT4 retention and release and thus controlling the flux of glucose over the plasma membrane of cells in rodents [13]. Although it is well established that TUG forms a complex with GLUT4 intracellularly, which is disrupted upon stimulation by insulin resulting in release of GLUT4 in rodents, less is known for human GLUT4 as the molecular details are not known.

**Fig. 5:**
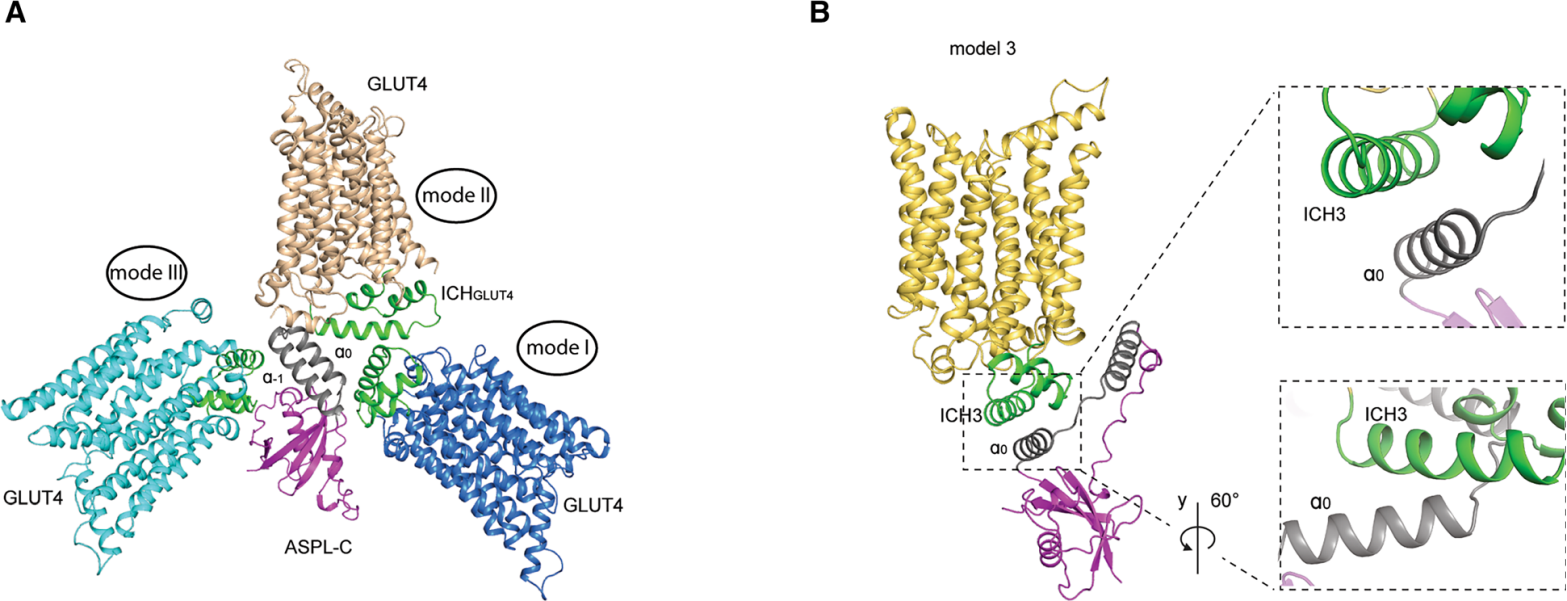
Docking models of GLUT4 (inward‐open, PDB ID: 7WSM) and ASPL‐C (PDB ID: 5IFS). (A) Cartoon representation of the three binding modes representing the nine docking models between GLUT4 and ASPL‐C by applying distance constraints. ICH domain in GLUT4 is colored in green and ASPL‐C is in same coloring strategy as previously. (B) Cartoon representation of model 3 (zoom‐out) and helical packing formed by ICH3 in GLUT4 and α_0_ helix in ASPL‐C shown in two different angles (zoom‐in). ICH domain in GLUT4 is colored in green and ASPL‐C is in same coloring strategy as previously.

Here we present evidence supporting that the human variant of TUG (ASPL) can form a complex with an intracellular domain of GLUT4. The XL‐MS data suggests that the intracellular domain of GLUT4 binds to the helical lariat of ASPL. Likewise, it has previously been reported that the interaction between TUG and GLUT4 in mouse involves the helical lariat (aa 313–376) in TUG [6] and likely the ICH domain in GLUT4 [12]. To analyze this further, the GLUT4 structure (PDB ID: 7WSM) was docked with ASPL‐C (PDB ID: 5IFS) globally by imposing distance constraints according to the identified crosslinking sites (Table 1), with the assumption that binding does not result in large conformational rearrangements in the binding partners upon complex formation. This resulted in nine different complex models with three overall binding modes (I, II, III; Fig. 5A, Fig. S4). ASPL is known to form a stable complex with p97 with an affinity of 0.2 nM [10], and as the vast majority of TUG is bound to p97 in cells [14] it is likely that the majority of ASPL is also bound to p97 in human cells. Hence, binding mode III is unlikely as p97 and GLUT4 are completely overlapping in the ternary model (Fig. S5). Interestingly, in the models displaying binding mode I, GLUT4 is docked in between the two long helices, α_−1_ and α_0,_ of the helical lariat of ASPL (Fig. S6). Since the lariat wraps around the N terminal domain of p97, this binding mode arranges GLUT4 and p97 in close proximity in the ternary model, suggesting that if this binding mode is viable there should be direct contacts between p97 and GLUT4. p97 controls numerous essential cellular processes and thus has several binding partners. This has been thoroughly examined by yeast two‐hybrid screen resulting in several high‐confidence p97 interaction partners including ASPL, UBX containing proteins, and also integral membrane proteins such as sodium‐independent purine‐selective nucleobase transporter (encoded by *SLC43A3*) [10]. However, GLUT4 was not detected as a p97 binding partner and has not been reported to be involved in forming a complex with p97 in other protein–protein interaction databases (https://string‐db.org/, or https://www.ebi.ac.uk/intact/home), indicating that binding mode I is not likely to happen *in vivo*. In contrast, the two models (number 3 and 5)  utilizing binding mode II display a conformation with direct contacts exclusively between ASPL and GLUT4, suggesting that these are plausible binding models (Fig. S7). This is supported by that all the identified cross‐links between ASPL and GLUT4 (Table 1) are < 30 Å apart in both models. In model 3 and 5, the GLUT4 ICH domain is in close proximity to the helical lariat of ASPL. To distinguish between the two models the buried surface area (BSA) was calculated by using PDBePISA server (https://www.ebi.ac.uk/pdbe/pisa/). The residues with BSA of ≥ 80 Å^2^ and/or those with accessible surface area (ASA) becoming buried to 85% or more upon complex formation [15] are listed in Table 2. Overall, model 3 has the larger BSA of the two models implying that this complex would have a higher affinity (Fig. S8). The main contact area in model 3 is between the ICH3 helix of GLUT4 and the α_0_ helix of ASPL forming a clear helix packing (Fig. 5B), which is in accordance with where the cross‐linked lysines were found (Fig. 4C). Moreover, helix packing is a well‐known mode of interaction observed in previously reported transmembrane protein complexes, suggesting that this is a plausible model [16].

**Table 2:** Analysis of buried surface areas (BSA). % represents the percentage of accessible surface area becoming buried upon complex formation; Å^2^ represents buried surface area.

	ASPL‐C (residue BSA)			GLUT4 (residue BSA)		
	Residues	%	Å^2^	Residues	%	Å^2^
Model 3	T373	100	66.8	Y231	100	19.52
	A375	100	71.26	R269	60	101.84
	A379	100	55.15			
Model 5	E368	80	84.75	R269	50	100.62

Previous data support the idea that other domains of the two proteins also contribute to complex formation [6, 12]. Interestingly, the unique sequence of the ICH3 helix of GLUT4, which contains only two conserved residues among GLUT1‐4 (both facing away from ASPL; Fig. S9) is the main contact area between ASPL and GLUT4. This observation may help to explain the GLUT isoform specificity of GLUT4 binding to ASPL. Our data also help to explain how insulin causes the release of GLUT4 from ASPL. The initial binding of GLUT4 to intact TUG/ASPL is likely cooperative, as evidence implies that there are at least two interacting regions on each protein [6, 10]. The binding affinity measured here will contribute to one of these, yet the overall interaction between the two proteins may have much higher affinity. In addition, TUG has been shown to bind insulin‐regulated membrane aminopeptidase (IRAP), a single transmembrane protein that co‐traffics with GLUT4 in the insulin‐responsive vesicles [17]. This interaction has been mapped to TUG residues 1–164 and IRAP residues 55–84, and it will also likely contribute to the ability of intact TUG/ASPL to trap the insulin‐responsive vesicles intracellularly. To release these vesicles, and to stimulate the dissociation of GLUT4 from intact TUG/ASPL, insulin has been shown to trigger endoproteolytic cleavage of TUG at the bond joining residues 164–165 [14]. GLUT4 binding to TUG/ASPL might then no longer be cooperative. After cleavage, GLUT4 and IRAP remain associated with the N‐terminal TUG cleavage product, which acts with molecular motors to carry the insulin‐responsive vesicles to the cell surface [14]. Dissociation of GLUT4 from the ASPL C‐terminal product may be facilitated by the reduced affinity of this interaction, compared with that involving full‐length TUG/ASPL.

In summary, herein, we report that ASPL is expressed and localized to the cytoplasm of human primary adipocytes and that it can form a complex with the intracellular domain of GLUT4 utilizing the helical lariat. Taken together the data supports the concept that ASPL can bind to GLUT4 in humans in similar manner as in rodents, and that it contributes to the regulation of GLUT4 trafficking upon insulin stimulation in human adipocytes.

## Experimental procedures

### Ethics

The study was approved by the Regional Ethics Committee, Regionala Etikprövningsnämnden in Lund, (Dnr 2017/920). All experiments were performed in accordance with relevant guidelines and regulations and after written informed consent obtained from all volunteers. Human abdominal subcutaneous adipose tissue was received from the Lund University hospital following the surgery in non‐diabetic female patients. The Declaration of Helsinki protocols were followed.

### Immunofluorescence of GLUT4 and ASPL in human adipocytes

Human primary adipocytes were isolated from surrounding tissue by collagenase digestion in a shaking incubator (37 °C, 170 r.p.m., 20 min) and filtering through cotton mesh. Adipocytes were washed in Krebs‐Ringer HEPES (KRH) buffer [118.6 mM NaCl, 4.7 mM KCl, 1.2 mM NaH_2_PO_4_, 1.2 mM MgSO_4_∙7H_2_O, 2.5 mM CaCl_2_∙2H_2_O, HEPES 25 mM pH 7.4, 2 mm glucose, 1% (w/v) bovine serum albumin (BSA), 200 nM adenosine] and PBS before being fixated in 4% paraformaldehyde (PFA). KRH buffer containing 2% (w/v) fish gelatin and 1 mg·mL^−1^ saponin was used for blocking and permeabilization for 10 min, 37 °C. Adipocytes were stained in scintillation vials (180 r.p.m., 1 h) with primary antibodies against GLUT4 (rabbit, polyclonal) [18] and ASPL (WH0079058M1, Sigma‐Aldrich, Saint Louis, MO, USA ) in KRH buffer. Secondary Alexa Fluor^®^ 568 conjugated (ab175470, Abcam, Cambridge, UK) and Alexa Fluor^®^ 488 conjugated (ab150105, Abcam, Cambridge, UK) antibodies were used under likewise conditions. Adipocytes were washed and transferred to mounting medium containing DAPI in a 35 mm round glass bottom petri dish with a 20 mm diameter imaging window. Microscopy was performed on a Nikon A1plus confocal microscope (Nikon Instruments, Amstelveen, The Netherlands) together with NIS‐elements, version: 4.50.02, by taking confocal stack images of adipocytes that were combined to form the full cell volume. Figures showing max intensity projection of fluorophores were prepared using ImageJ version: 2.9.0/1.53 t [19].

### Immunogold electron microscopy of human adipose tissue

The adipose tissue was fixed in 4% formaldehyde and 0.1% glutaraldehyde, then embedded and subjected to preparation of ultrathin sections. Immunogold labeling of adipose tissue was performed as described previously with minor modification [20]. Briefly, specimens were embedded in methacrylate resin (Lowicryl HM20, Polysciences, Warrington, PA, USA) and polymerized by UV light below 0 °C. Ultrathin sections were cut using an Ultratome (Reichert Ultracut S, Leica, Wetzlar, Germany) and placed on 300 mesh copper grids. Three sections selected blindly from three individuals were rinsed in TBST containing 50 mM glycine, incubated in 2% human serum albumin (HSA), followed by incubation with either GLUT4 antibody [18] or ASPL antibody (WH0079058M1, Sigma‐Aldrich, Saint Louis, MO, USA) overnight, followed by incubation with corresponding secondary antibody conjugated with 15 nm colloidal gold (Abcam, Cambridge, UK) for 60 min, and contrasted with 2% uranyl acetate for 10 min and 0.3% lead citrate for 2 min. The sections were examined using a Tecnai 12 transmission electron microscope at 80 kV.

### Proximity ligation assay of GLUT4 and ASPL in human adipocytes

Human primary adipocytes were isolated, fixed, and prepared for staining as previously described. Likewise, cells were labeled with primary antibodies against GLUT4 (rabbit, polyclonal) [18] and ASPL (WH0079058M1, Sigma‐Aldrich, Saint Louis, MO, USA). For the proximity ligation (PLA) assay Duolink *In Situ* reagents (Sigma‐Aldrich) were used together with Duolink Fluorescent Detection Reagents Orange (Sigma‐Aldrich, Saint Louis, MO, USA), following manufacturer's instructions. Briefly, 50 μL densely packed, antibody labeled adipocytes were washed in blocking buffer (KRH with 2% fish gelatin and 1 mg·mL^−1^ saponin) followed by incubation (37 °C, 170 r.p.m., 1 h) with PLA probe anti‐rabbit MINUS to detect GLUT4 primary antibody and PLA probe anti‐mouse PLUS to detect ASPL primary antibody. After several washing steps in provided washing buffer, the adipocytes were incubated with a ligation solution containing oligonucleotides and ligase (37 °C, 30 min) with several inversions of the tube for mixing during incubation. After subsequent washing steps an amplification solution with polymerase and fluorophore‐labeled oligos was added (37 °C, 100 min). After multiple washing steps, the adipocytes were transferred to glass bottom dishes with mounting medium containing DAPI. They were visualized on a Nikon A1plus confocal microscope (Nikon Instruments, Amstelveen, The Netherlands) with NIS‐elements, version: 4.50.02. Stack images were made into maximum intensity projections in ImageJ version: 2.9.0/1.53 t. Interaction points were changed from red to yellow color to make them more visible in print.

### GLUT4 expression and lysate preparation

Rat GLUT4 with N‐terminal poly‐histidine tag and human AQP7 (Aquaporin 7, as a control in far dot western blot) with C‐terminal His‐tag were cloned into the double‐deletion strain *Pichia pastoris* GS115 aqy1Δ::HIS4 agp1Δ::NatMX with no endogenous aquaporins and aquaglyceroporins present [21]. The yeast cells with GLUT4 and AQP7 construct were cultured as previously described [22]. Harvested cells were lysed with high‐pressure homogenization apparatus (Biox AB, Onsala, Sweden), subsequently, membranes were isolated by ultra‐centrifugation at 145 000 x *g* for 90 min at 4 °C and solubilized in 20 mM sodium phosphate pH 8.0, 300 mM NaCl, 10% glycerol, 1 mM DTT, EDTA‐free protease inhibitor cocktail (Merck, Darmstadt, Germany) and 1% decyl β‐D‐maltopyranoside (DM, Anatrace, Maumee, OH, USA) for 1 h at 4 °C. Homogenate was clarified by centrifugation at 145 000 x *g* for 30 min, and subjected to western blot analysis. In brief, the PVDF membrane was blocked in 2% fish gelatin before incubating with GLUT4 antibody [18] and corresponding HRP conjugated secondary antibody (Invitrogen, Waltham, MA, USA) to identify GLUT4 protein in the lysate. Finally, the signal was recorded with Odyssey Fc gel‐scanning system (LI‐COR, Lincoln, NE, USA).

### Protein expression and purification

GST‐tagged human full‐length ASPL with C‐terminal His tag and GST‐tagged ASPL‐C including residues 313–553 (both ASPL plasmids were received as gift from Erich E, Wanker in Germany) were expressed in *E. coli* strain BL21 DE3 Rosetta2 (Invitrogen, Waltham, MA, USA) with induction of 1 mM isopropyl‐b‐D‐thio‐galactopyranoside (IPTG, Thermo Fisher, Waltham, MA, USA) and purified as described previously with minor modification [10]. Briefly, *E. coli* cell pellets containing ASPL and ASPL‐C were resuspended in PBS and supplemented with EDTA‐free protein inhibitor cocktail (Roche, Basel, Switzerland), DNaseI (Roche, Basel, Switzerland) and 1 mM DTT were disrupted by brief sonication, respectively. Subsequently, cell debris were clarified by centrifugation and supernatants were incubated with glutathione‐Sepharose 4B beads (Invitrogen, Waltham, MA, USA) overnight before beads were washed with PBS and proteins were eluted with 30 mM reduced glutathione in PBS. ASPL protein was further purified with Cobalt resin (Thermo Scientific, Rockford, IL, USA) and eluted with 300 mM imidazole in PBS buffer. GST tag is cleavable by TEV according to further experimental requirement. Concentrated ASPL‐C proteins was loaded to Superdex 75 increase 10/300 column (GE Healthcare, Chicago, IL, USA) equilibrated with PBS buffer, and eluted peak fractions were pooled together, concentrated and flash frozen until further use. Finally, protein purity was analyzed by Coomassie‐stained SDS/PAGE.

The genes coding for thioredoxin (Trx) and Trx fused C‐terminally with human GLUT4 intracellular helix bundle (residues 225–271) with substitutions of I232C and K264C, were synthesized (GenScript, Piscataway, NJ, USA) and cloned into pET24a vector and expressed in *E. coli* strain TUNER (DE3) with induction of 1 mM IPTG. Harvested cells were resuspended in PBS and supplemented with EDTA‐free protein inhibitor cocktail (Roche, Basel, Switzerland) and DNaseI (Roche, Basel, Switzerland), followed by sonication. After removing cell debris by centrifugation, supernatants were incubated with nickel‐affinity resin (Ni‐NTA agarose, Qiagen, Hilden, Germany) for batch affinity chromatography. Proteins were eluted with 300 mm imidazole, concentrated and subsequently applied to a size‐exclusion chromatography column (Superdex 75 increase 10/300).

### Pull‐down assay

GLUT4 lysate was prepared from yeast cell membranes by detergent solubilization and ultracentrifugation as described above. The lysate was incubated with ASPL immobilized glutathione‐Sepharose 4B beads, or only beads (as a control) for 1 h at 4 °C before washing with PBS. Putative bound proteins were eluted with 30 mM reduced glutathione in PBS and subjected to western blot analysis with specific GST antibody (Thermo Fisher) and GLUT4 antibody [18].

10 μg Trx‐ICH_GLUT4_ and Trx (as a control) were immobilized on nickel‐affinity resin (Ni‐NTA agarose, Qiagen), respectively, before incubation with ASPL‐C protein overnight at 4 °C. The resin was washed with PBS, and the protein bound to the beads was eluted using 300 mM imidazole in PBS. The eluted fractions were identified and analyzed by SDS/PAGE and western blotting.

### Far dot western blot

Far dot western blotting was carried out as previously described with minor modifications [23]. Briefly, 1 μL of ASPL‐C and PcoC (copper transporter expressed in *E. coli* [24], as a control) containing 100 pmol protein were spotted onto nitrocellulose membranes, before blocking in 2% fish gelatin and 1% BSA for 40 min. GLUT4 and AQP7 (as a control) lysates were prepared as described above and applied to membrane at room temperature for 40 min before washing twice with PBS supplemented with 0.1% DM. Next, membranes were incubated with GLUT4 antibody [18], AQP7 antibody (ab32826, Abcam, Cambridge, UK) subsequently with corresponding HRP conjugated secondary antibody (Invitrogen, Waltham, MA, USA). The signals were recorded using an Odyssey Fc gel‐scanning apparatus (LI‐COR, Lincoln, NE, USA). All images were applied to background correction with rolling‐ball filter in ImageJ, and the intensity of the signal was quantified using imagej.

### Surface plasmon resonance (SPR)

His‐tagged Trx‐ICH_GLUT4_, Trx and ASPL‐C were prepared as described above. ASPL‐C in 10 mm sodium acetate (pH 5.0) was immobilized onto the CM5 chip (GE Healthcare) at 1000 response units (RU) via amine coupling. Trx‐ICH_GLUT4_ and Trx were diluted into concentration series ranging from 0.6 to 83 μm in running buffer (10 mM HEPES, 150 mM NaCl, 0.005% Tween20, pH 7.4) respectively, before injecting over immobilized ASPL‐C. Experiments were performed on a BIAcore 3000 system and run in duplicates. RU were recorded against time (s) for each concentration and replotted in GraphPad Prism 9.5.1 (GraphPad Software, San Diego, CA, USA). Only reference subtracted data were considered. Data points in duplicates were shown and binding curve was fitted by using nonlinear regression in GraphPad Prism. Equilibrium disassociation constants (*K*_D_) for Trx‐ICH_GLUT4_ and ASPL‐C complex was determined by one‐site specific binding model with eq. *Y* = *B*_max_ * *X*/(*K*_D_ + *X*) in which *Y* is response units (RU) while *X* is the concentration of Trx‐ICH_GLUT4_ as analyte. Bmax represents the extrapolated maximum binding when the interaction sites become saturated.

### Cross‐linking mass spectrometry (XL‐MS)

For cross‐linking, 5 μg of purified Trx‐ICH_GLUT4_ was incubated with ASPL‐C in a final volume of 50 μL for 15 min at 37 °C, 500 r.p.m. To crosslink the proteins, heavy/light disuccinimidyl suberate crosslinker (DSS‐H12/D12, Creative Molecules Inc., San Fransisco, CA, USA) resuspended in 100% dimethylformamide (DMF) was added to final concentrations of 0, 0.25, 0.5, 1 and 2 mM, and incubated for 30 min at 37 °C, 800 r.p.m. The reaction was quenched by ammonium bicarbonate at a final concentration of 50 mm followed by incubation for 15 min at room temperature. For mass spectrometry, the crosslinked proteins were denatured with 4 m urea and 100 mm ammonium bicarbonate, and the disulphide bonds were reduced with a final concentration of 5 mm Tris (2‐carboxyethyl) phosphine hydrochloride (TCEP) for 60 min at 37 °C, 800 r.p.m., and alkylated with a final concentration of 10 mm iodoacetamide for 30 min in the dark at room temperature. The proteins were digested with 1 μg of lysyl endopeptidase (Wako Chemicals, Richmond, VA, USA) for 2 h at 37 °C, 800 r.p.m., subsequently, samples were diluted to a final urea concentration < 1.5 m with 100 mm ammonium bicarbonate, followed by further digestion using 1 μg sequencing grade trypsin (Promega, Madison, WI, USA) for 18 h at 37 °C, 800 r.p.m. The digested samples were acidified with 10% formic acid to pH 3. Peptides were purified and desalted using C18 reverse phase spin columns according to the manufacturer's instructions (Macrospin columns, Harvard Apparatus, Holliston, MA, USA). Peptides were dried and then reconstituted in 2% acetonitrile, 0.2% formic acid before analysis on Q Exactive HF‐X mass spectrometer (Thermo Scientific) connected to an EASY‐nLC 1200 ultra‐high‐performance liquid chromatography system (Thermo Scientific). The peptides were loaded onto an Acclaim PepMap 100 (75 μm × 2 cm) C18 (3 μm, 100 Å) pre‐column and separated on an EASY‐Spray column (Thermo Scientific; ID 75 μm × 50 cm, column temperature 45 °C) operated at a constant pressure of 800 bar. One full MS scan (resolution 60 000 at 200 *m*/*z*; mass range 390–1210 *m*/*z*) was followed by MS/MS scans (resolution 15 000 at 200 *m*/*z*) of the 15 most abundant ion signals. The isolation width window for the precursor ions was 2 *m*/*z*, and they were fragmented using higher‐energy collisional‐induced dissociation (HCD) at a normalized collision energy of 30. Charge state screening was enabled, and precursor ions with unknown charge states and a charge state of 1 were rejected. The dynamic exclusion window was 10 s. The automatic gain control was set to 3e6 and 1e5 for MS and MS/MS with ion accumulation times of 110 and 60 ms, respectively. The intensity threshold for precursor ion selection was set to 1.7e4. All samples were analyzed using MeroX (version 2.0.0.8) [25] and pLink 2  (version 2.3.9) [26]. The target protein database contained the Trx‐ICH_GLUT4_ and ASPL‐C sequences. pLink2 was run using default settings for DSS‐H12/D12 cross‐linking, with trypsin as the protease and up to 3 missed cleavages allowed. Peptides with a mass range of 600–6000 *m*/*z* were selected, and the precursor and fragment tolerance were set to 10 and 20 ppm, respectively. The mass spectrometry data has been deposited to the ProteomeXchange [27] consortium via the MassIVE partner repository (https://massive.ucsd.edu/) with the dataset identifier PXD041130.

### Model docking

Global docking was run for ICH_GLUT4_ and ASPL‐C using EvoDOCK [28]. EvoDOCK is an all‐atom protein–protein docking method that involves rigid‐body and side‐chain optimization, with sampling of backbone changes via conformational ensembles. Simulations were carried out as described in Varela et al. [28], but with an additional modification to drive the docking orientations towards solutions compatible with the mass spectrometry data. To this end, the docking simulations were guided by the Rosetta energy function [29] + (constraint energy term * 10/100). Each experiment was performed by 100 independent runs; thus 100 different “optimized” models were generated from each experiment. However, no relevant difference was identified between the simulations when the constraint energy term multiplied by 10 or 100. Final models were filtered by the distances between each pair of crosslinking amino acids below 33 Å, resulting in nine different models.

## Conflict of interest

The authors declare no conflict of interest.

### Peer review

The peer review history for this article is available at https://www.webofscience.com/api/gateway/wos/peer‐review/10.1002/2211‐5463.13709.

## Author contributions

PH and KL‐P conceptualization; KL‐P project administration; PG, MRA‐M, IA and KL‐P supervision; PH, JSB, PG, MRA‐M, IA and KL‐P funding acquisition; PG, MRA‐M, IA and KL‐P resources; PH, HÅ, DV, RV and LH investigation; PH, HÅ, DV, RV and LH formal analysis; PH and KL‐P writing–original draft; PH, HÅ, DV, RV, LH, JSB, PG, MRA‐M, IA and KL‐P writing–reviewing and editing.

## Supporting information


**Fig. S1.** Sequence alignment between ASPL (human) and TUG (mouse).
**Fig. S2.** Maximum intensity projections of human adipocyte confocal microscopy images. Magnification is 20x. Scale bars are 50 μM. (A) Adipocyte expression of ASPL (green) and GLUT4 (red). Nuclei stained with DAPI (blue). (B) Staining controls showing adipocytes without primary antibody to GLUT4 (left) or to ASPL (right).
**Fig. S3.** Surface plasmon resonance (SPR) analysis between ASPL‐C and Trx‐ICHGLUT4. A two‐fold concentration series of ICHGLUT4 ranging from 0.6 μM to 83 μM was injected over ASPL‐C immobilized on the CM5 chip, performed in duplicates. The response (response unit, RU) at t = 223 s is shown against concentrations, and data points in duplicates are shown and fitted with curve by nonlinear regression. The KD value is calculated via nonlinear regression in GraphPad Prism as well. After calculation, KD = 68 μM with 95% CI (Confidence Interval, 50 to 102), Bmax = 286 with 95% CI (Confidence Interval, 224 to 401).
**Fig. S4.** The nine docking models shown as cartoon. The models are classified into three binding modes (I, II, II), and colored accordingly in scales, blue, yellow, green, respectively. ASPL‐C is colored by magenta with helical lariat in gray.
**Fig. S5.** The binding mode III as cartoon showing steric clash between GLUT4 and p97. GLUT4 in cyan and P97 in dark green. ASPL‐C is colored by magenta with helical lariat in gray.
**Fig. S6.** Cartoon representation of mode I with GLUT4 in blue, P97 is in dark green, and ASPL‐C is colored by magenta with helical lariat in gray.
**Fig. S7.** Cartoon representation of model 3 and 5. (A) Model 3 with GLUT4 in yellow, and (B) model 5 with GLUT4 in brown. P97 is in dark green, and ASPL‐C is colored by magenta with helical lariat in gray.
**Fig. S8.** Surface representation of helical lariat (α0 and α‐1) in ASPL‐C. The key residues contributing BSA on the interface between two proteins in models 3 and 5 are colored by yellow and brown, respectively.
**Fig. S9.** The unique property of ICH3 domain in human GLUT4. (A) The sequence alignment for ICH3 domain in human GLUT1‐4. The conserved E259 and E263 are highlighted in red shadow. (B) Human GLUT4 ICH3 domain in docking model 3 (zoomout) showing these two conserved glutamate residues facing away from α0 helix in ASPL‐C.

## Data Availability

The mass spectrometry data has been deposited to the ProteomeXchange consortium via the MassIVE partner repository (https://massive.ucsd.edu/) with the dataset identifier PXD041130. All other data are contained within the manuscript and the supporting information.
