# Supplementary material for: The intracellular helical bundle of human glucose transporter GLUT4 is important for complex formation with ASPL
Source: FEBS Open Bio. 2023 Sep 28;13(11):2094–107. doi: 10.1002/2211-5463.13709 (PMC10626271; doi:10.1002/2211-5463.13709)
Supplement: Supplementary file 1 — Fig. S1. Sequence alignment between ASPL (human) and TUG (mouse). Fig. S2. Maximum intensity projections of human adipocyte confocal microscopy images. Magnification is 20x. Scale bars are 50 μM. (A) Adipocyte expression of ASPL (green) and GLUT4 (red). Nuclei stained with DAPI (blue). (B) Staining controls showing adipocytes without primary antibody to GLUT4 (left) or to ASPL (right). Fig. S3. Surface plasmon resonance (SPR) analysis between ASPL‐C and Trx‐ICHGLUT4. A two‐fold concentration series of ICHGLUT4 ranging from 0.6 μM to 83 μM was injected over ASPL‐C immobilized on the CM5 chip, performed in duplicates. The response (response unit, RU) at t = 223 s is shown against concentrations, and data points in duplicates are shown and fitted with curve by nonlinear regression. The KD value is calculated via nonlinear regression in GraphPad Prism as well. After calculation, KD = 68 μM with 95% CI (Confidence Interval, 50 to 102), Bmax = 286 with 95% CI (Confidence Interval, 224 to 401). Fig. S4. The nine docking models shown as cartoon. The models are classified into three binding modes (I, II, II), and colored accordingly in scales, blue, yellow, green, respectively. ASPL‐C is colored by magenta with helical lariat in gray. Fig. S5. The binding mode III as cartoon showing steric clash between GLUT4 and p97. GLUT4 in cyan and P97 in dark green. ASPL‐C is colored by magenta with helical lariat in gray. Fig. S6. Cartoon representation of mode I with GLUT4 in blue, P97 is in dark green, and ASPL‐C is colored by magenta with helical lariat in gray. Fig. S7. Cartoon representation of model 3 and 5. (A) Model 3 with GLUT4 in yellow, and (B) model 5 with GLUT4 in brown. P97 is in dark green, and ASPL‐C is colored by magenta with helical lariat in gray. Fig. S8. Surface representation of helical lariat (α0 and α‐1) in ASPL‐C. The key residues contributing BSA on the interface between two proteins in models 3 and 5 are colored by yellow and brown, respectively. Fig. S9. The [file FEB4-13-2094-s001.pdf]

Supplementary figures for

The intracellular helical bundle of human glucose transporter GLUT4 is important for complex formation with ASPL

Peng Huang[1], Hannah Åbacka[1], Daniel Varela[2], Raminta Venskutonytė[1,6], Lotta Happonen[3], Jonathan S Bogan[4], Pontus Gourdon[1], Mahmood R Amiry-Moghaddam[5], Ingmar André[2], Karin Lindkvist-Petersson[1, 6].

1. Department of Experimental Medical Science, Lund University, 22 184, Lund, Sweden
2. Department of Biochemistry and Structural Biology, Lund, University, PO Box 124, SE-221 00, Lund, Sweden.
3. Division of Infection Medicine, Department of Clinical Sciences Lund, Lund University, 22 184 Lund, Sweden
4. Section of Endocrinology and Metabolism, Department of Internal Medicine, and Department of Cell Biology, Yale School of Medicine, New Haven, CT, United States.
5. Laboratory of Molecular Neuroscience, Division of Anatomy, Department of Molecular Medicine, Institute of Basic Medical Sciences, University of Oslo, Post box 1105, Blindern, 0317 Oslo, Norway
6. LINXS - Lund Institute of Advanced Neutron and X-ray Science, Lund, Sweden.

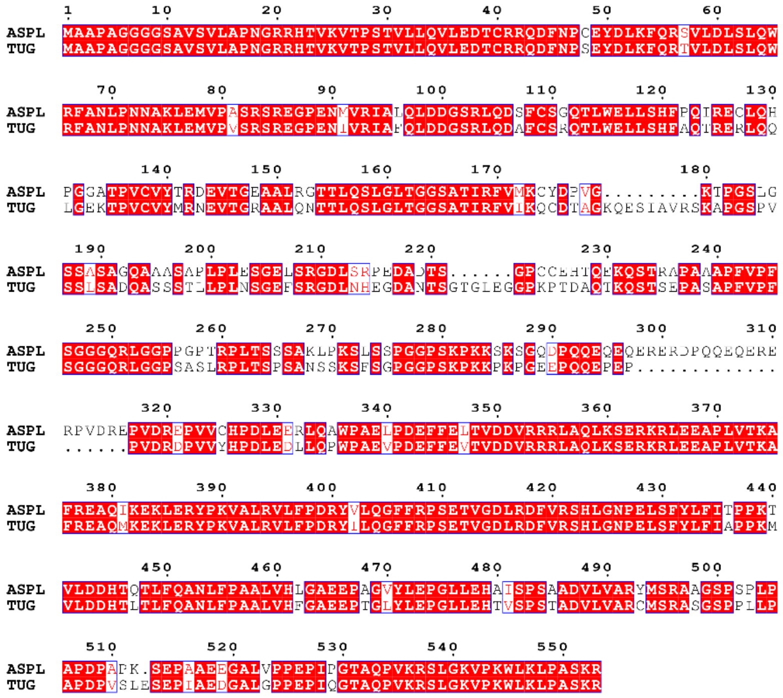

Supplementary Fig. 1 Sequence alignment between ASPL (human) and TUG (mouse).

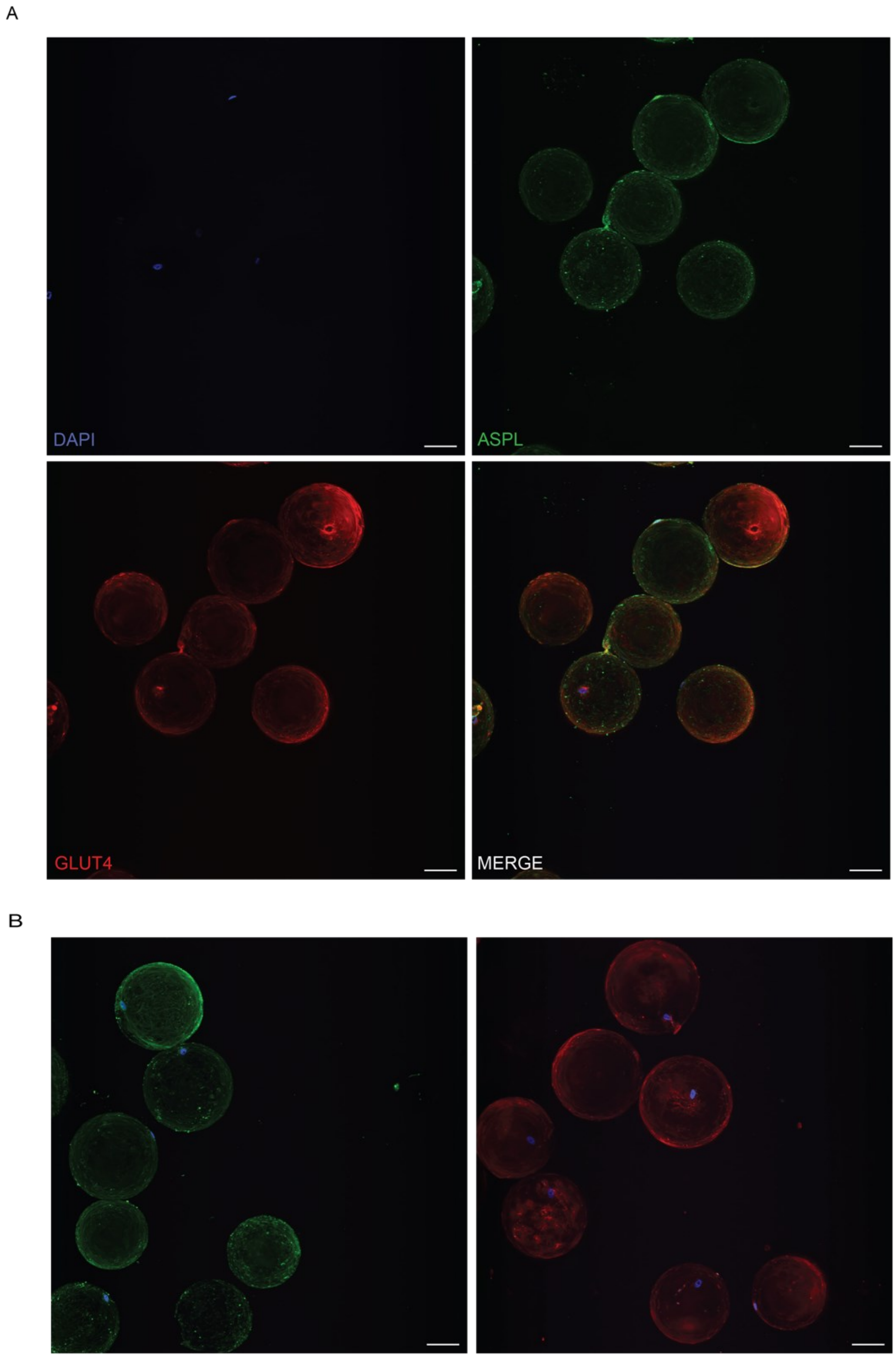

Supplementary Fig. 2 Maximum intensity projections of human adipocyte confocal microscopy images. Magnification is 20x. Scale bars are 50 µM. (A) Adipocyte expression of ASPL (green) and GLUT4 (red). Nuclei stained with DAPI (blue). (B) Staining controls showing adipocytes without primary antibody to GLUT4 (left) or to ASPL (right).

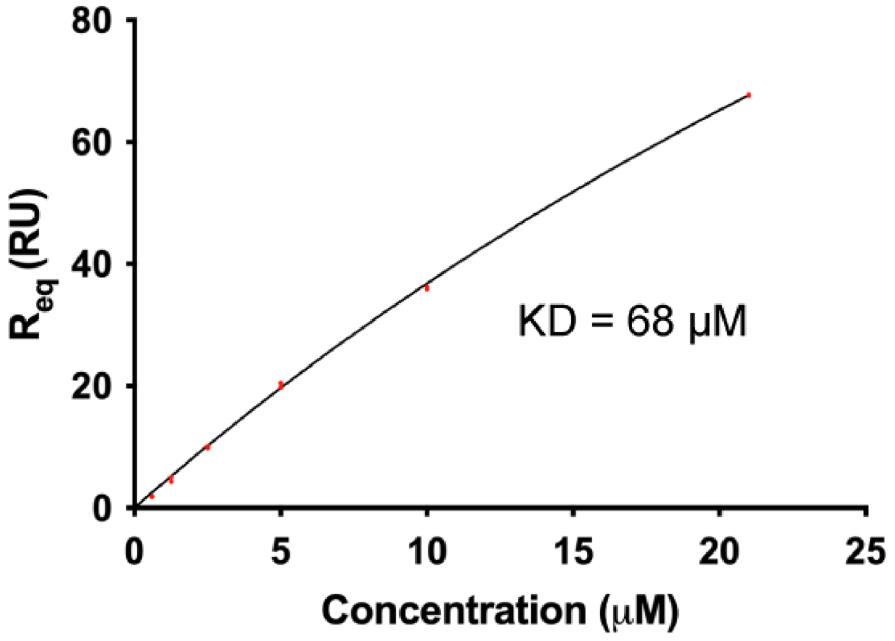

Supplementary Fig. 3 Surface plasmon resonance (SPR) analysis between ASPL-C and Trx-ICH_{GLUT4}. A two-fold concentration series of ICH_{GLUT4} ranging from 0.6 µM to 83 µM were injected over ASPL-C immobilized on the CM5 chip, performed in duplicates. The response (response unit, RU) at t=223 s is shown against concentrations, and data points in duplicates are shown and fitted with curve by nonlinear regression. The K_D value is calculated via nonlinear regression in GraphPad Prism as well. After calculation, KD = 68 µM with 95% CI (Confidence Interval, 50 to 102), Bmax = 286 with 95% CI (Confidence Interval, 224 to 401)

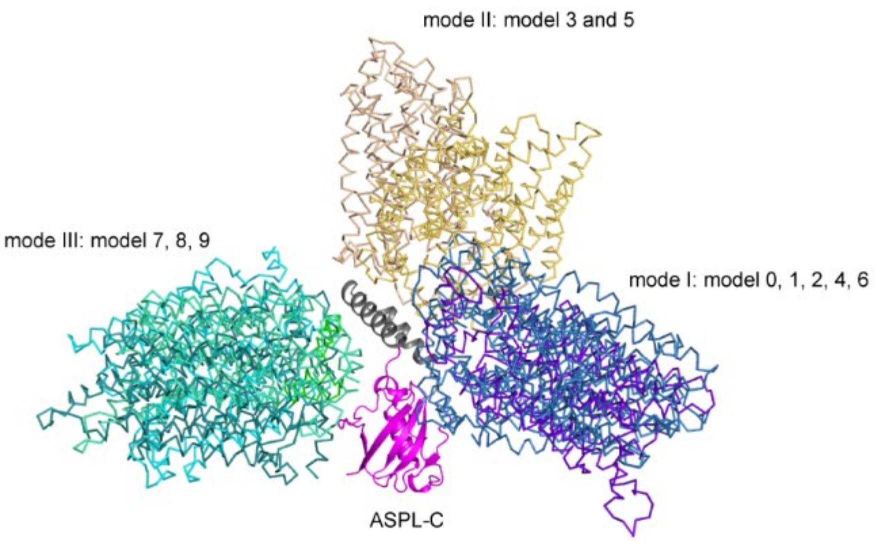

Supplementary Fig. 4 The nine docking models shown as cartoon. The models are classified into three binding modes (I, II, II), and colored accordingly in scales, blue, yellow, green, respectively. ASPL-C is colored by magenta with helical lariat in gray.

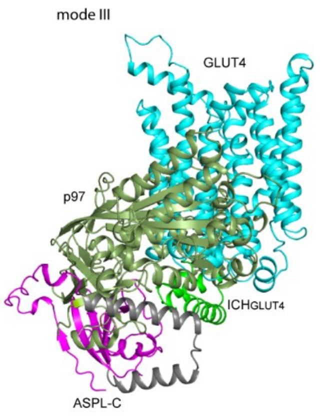

Supplementary Fig. 5 The binding mode III as cartoon showing steric clash between GLUT4 and p97. GLUT4 in cyan and P97 in dark green. ASPL-C is colored by magenta with helical lariat in gray.

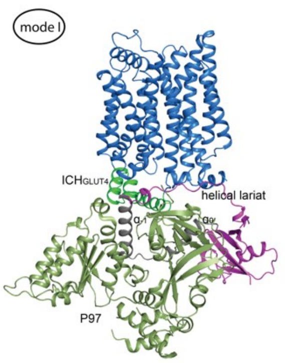

Supplementary Fig. 6 Cartoon representation of mode I with GLUT4 in blue, P97 is in dark green, and ASPL-C is colored by magenta with helical lariat in gray.

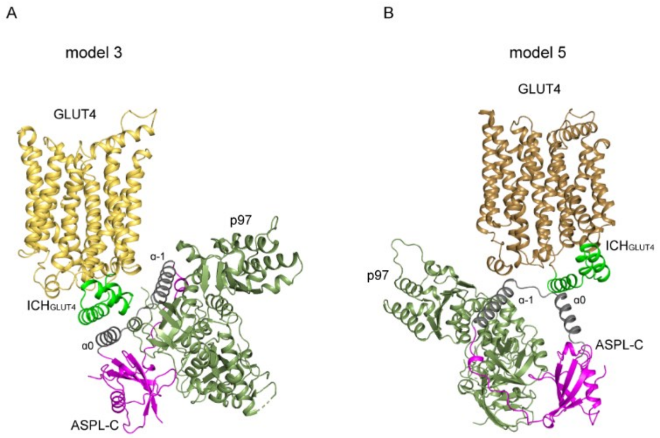

Supplementary Fig. 7 Cartoon representation of model 3 and 5. (A) Model 3 with GLUT4 in yellow, and (B) model 5 with GLUT4 in brown. P97 is in dark green, and ASPL-C is colored by magenta with helical lariat in gray.

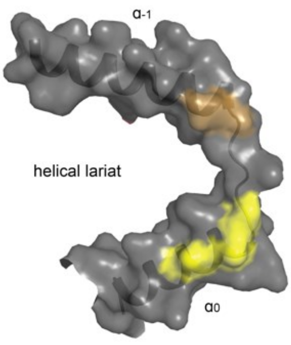

Supplementary Fig. 8 Surface representation of helical lariat (α_0 and α_{-1}) in ASPL-C. The key residues contributing BSA on the interface between two proteins in models 3 and 5 are coloured by yellow and brown, respectively.

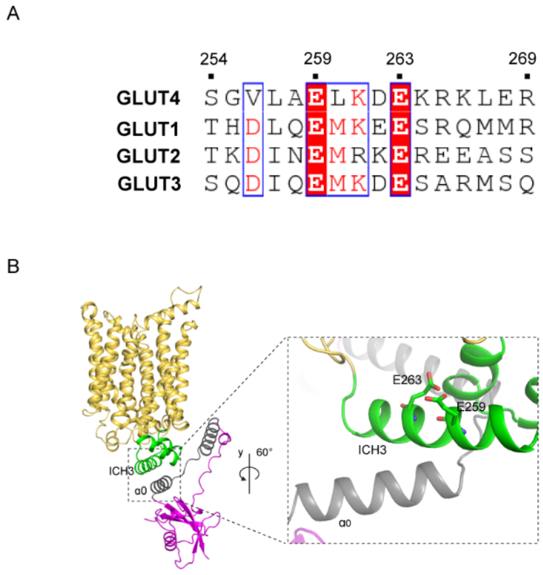

Supplementary Fig. 9 The unique property of ICH3 domain in human GLUT4. (A) The sequence alignment for ICH3 domain in human GLUT1-4. The conserved E259 and E263 are highlighted in red shadow. (B) Human GLUT4 ICH3 domain in docking model 3 (zoom-out) showing these two conserved glutamate residues facing away from α_0 helix in ASPL-C
